# Infektionsgeschehen und Eindämmungsmaßnahmen in Kitas während der COVID-19-Pandemie – Erkenntnisse aus der Corona-KiTa-Studie

**DOI:** 10.1007/s00103-021-03449-z

**Published:** 2021-11-03

**Authors:** Julika Loss, Susanne Kuger, Udo Buchholz, Ann-Sophie Lehfeld, Gianni Varnaccia, Walter Haas, Susanne Jordan, Bernhard Kalicki, Anja Schienkiewitz, Thomas Rauschenbach

**Affiliations:** 1 Abteilung für Epidemiologie und Gesundheitsmonitoring, Robert Koch-Institut, General-Pape-Str. 62–66, 12101 Berlin, Deutschland; 2 Zentrum für Dauerbeobachtung und Methoden, Deutsches Jugendinstitut, München, Deutschland; 3 Abteilung für Infektionsepidemiologie, Robert Koch-Institut, Berlin, Deutschland; 4 Abteilung Kinder und Kinderbetreuung, Deutsches Jugendinstitut, München, Deutschland; 5 Institutsleitung, Deutsches Jugendinstitut, München, Deutschland

**Keywords:** Kindertagesbetreuung, SARS-CoV-2-Ausbruch, COVID-19, Inzidenz, Sekundäre Attack-Rate, Children daycare center, SARS-CoV‑2 outbreak, COVID-19, Incidence, Secondary attack rate

## Abstract

**Hintergrund:**

Kindertageseinrichtungen (Kitas) spielen eine wichtige gesellschaftspolitische Rolle; gleichzeitig begünstigt der enge Kontakt der Kinder in Kitagruppen untereinander und mit Beschäftigten die Übertragung von Infektionen. In der COVID-19-Pandemie ergaben sich die Fragen, wie Infektionsgeschehen in Kitas verlaufen, welche Rolle Kitakinder in der Pandemie spielen und welche Schutz- und Hygienemaßnahmen in Kitas umgesetzt werden. Von 06/2020 bis 12/2021 wird die „Corona-KiTa-Studie“ durchgeführt, in der pädagogische und infektionsepidemiologische Themen gemeinsam bearbeitet werden.

**Methoden:**

In der Studie werden Daten aus unterschiedlichen Quellen erhoben. Es werden amtliche Meldedaten sowie wöchentliche Angaben von Kitas im sog. KiTa-Register kontinuierlich ausgewertet. Zudem werden SARS-CoV-2-Ausbrüche in Kitas vor Ort durch wiederholte Probengewinnung und Befragungen untersucht.

**Ergebnisse:**

Das SARS-CoV-2-Infektionsgeschehen in Kitas bzw. bei Kindern im Kitaalter war von 03/2020 bis 05/2021 sehr dynamisch. In der 2. und 3. Pandemiewelle stiegen SARS-CoV-2-Ausbrüche in Kitas deutlich an, begleitet von einer erheblichen Zunahme an Kita- und Gruppenschließungen. Zuletzt erhöhte sich der Anteil der betroffenen Kinder bei den Ausbrüchen kontinuierlich. Allerdings ergab die erste Untersuchung von SARS-CoV-2-Ausbrüchen (*n* = 28), dass bei kindlichen Indexfällen im Schnitt nur ein Bruchteil der Kitakontakte (6,8 %) angesteckt wurde. Die Übertragungshäufigkeiten unterschieden sich zwischen einzelnen Kitas deutlich.

**Diskussion:**

Die Zusammenführung von regelmäßig erhobenen Melde- und Befragungsdaten sowie Ausbruchsuntersuchungen ermöglicht ein vielschichtiges Monitoring des Infektionsgeschehens in Kitas, dessen Ergebnisse in Empfehlungen für Public-Health-Maßnahmen einfließen können.

## Abstract (en)

**Background:**

Daycare centers are of substantial sociopolitical and pedagogical relevance; at the same time, the close contact of children in daycare groups among each other and with employees favors the transmission of infections. In the COVID-19 pandemic, questions arose about how infection events occur in daycare centers, what role daycare children play in the pandemic, and what protective and hygienic measures are implemented in daycare centers. From 06/2020 to 12/2021, we conducted the “Corona Day Care Study,” in which we address pedagogical and infection epidemiological topics in a joint approach.

**Methods:**

In the study, data are collected from different sources. Official reporting data as well as weekly data from daycare centers in the so-called KiTa Register are continuously evaluated. In addition, SARS-CoV‑2 outbreaks in daycare centers are investigated on site by repeated sample collection and interviews.

**Results:**

SARS-CoV‑2 infection incidence in daycare centers or in daycare-aged children was very dynamic from 03/2020 to 05/2021. In the second and third pandemic waves, the number of SARS-CoV‑2 outbreaks in daycare centers rose sharply, accompanied by a substantial increase in daycare and group closures. Most recently, the proportion of affected children in outbreaks increased steadily. However, preliminary examinations of SARS-CoV‑2 outbreaks (*n* = 28) revealed that, on average, only a fraction of daycare contact persons (6.8%) were infected by child index cases. Transmission frequencies differed markedly between the individual daycare centers.

**Discussion:**

The combination of regularly collected reporting and survey data as well as outbreak investigations allows a multilayered monitoring and understanding of infection events in daycare centers; its findings could be incorporated into recommendations for public health measures.

## Einleitung

### Kitas als möglicher Ort für SARS-CoV-2-Übertragungen

Kindertageseinrichtungen bzw. -tagespflege (zusammen: Kitas) spielen eine zentrale Rolle in der Bildungsbiografie von Kindern. Zugleich ist die Kindertagesbetreuung für viele Eltern die einzige Möglichkeit, Beruf und Familie zu vereinbaren. Der Besuch einer Kita vor der Einschulung gehört für Kinder mittlerweile zur Normalität. In den letzten Jahren ist die Zahl an Kindern, die ein Kindertagesbetreuungsangebot nutzten, kontinuierlich gestiegen. Im Jahr 2020 waren dies 35 % der unter 3‑Jährigen sowie 92,8 % der 3‑ bis 6‑jährigen Kinder. Damit wurden etwa 3,4 Mio. Kinder in einer der ca. 52.870 Kindertageseinrichtungen in Deutschland betreut [1, 2]. In 2020 arbeiteten in allen Kindertageseinrichtungen zusammengenommen mit Hort ca. 785.700 Beschäftigte, davon ca. 675.645 als pädagogisches oder leitendes Personal; hinzu kommen ca. 44.800 Personen, die in der Kindertagespflege tätig sind [3].

Die Kindertagesbetreuung ist auch in den Fokus von Public Health gerückt. Zum einen stellen Kitas eine Lebenswelt von Kindern dar, in der sie viel Zeit verbringen und die dadurch z. B. ihr Bewegungs- und Ernährungsverhalten beeinflusst. Kitas stellen somit ein sogenanntes Setting dar, das sich für präventive Lebensstilinterventionen anbietet [4]. Zum anderen erhöht das enge Zusammensein in Gruppen, wie es für Kitas charakteristisch ist, das Übertragungsrisiko bestimmter viraler und bakterieller Infektionskrankheiten und kann damit Krankheitsausbrüche begünstigen, z. B. durch gastrointestinale Infekte mit Rotaviren oder respiratorische Erkrankungen durch Rhino- oder RS-Viren [5, 6]. Für mehrere Infektionskrankheiten gilt, dass ihr Vorkommen in der Allgemeinbevölkerung oft mit Infektionsgeschehen in Kitas assoziiert ist [6].

Eine Datenerhebung aus Kitas kann daher gut als ein Frühindikator für (kommende) Entwicklungen in der Allgemeinbevölkerung dienen. Hierfür eignen sich spezifische Indikatoren, wie die Erfassung laborbestätigter Infektionen, z. B. über das amtliche Meldesystem, oder weniger spezifische, aber sensitivere Indikatoren, die Symptome bzw. Syndrome oder Abwesenheiten erfassen und damit ermöglichen, die generelle Dynamik oder gar Ausbruchsgeschehen frühzeitig zu erkennen [7, 8]. Da Übertragungen in Kitas eine Epidemie verstärken (amplifizieren) können, wird z. B. in Hinblick auf Eindämmungsmaßnahmen bei Influenzaepidemien empfohlen, die Schließung von Kitas zu erwägen [9–11]. Dieses Vorgehen ist allerdings nicht ohne Weiteres auf andere Ausbrüche durch Viren, z. B. SARS-CoV‑2, übertragbar, da stets auch das jeweilige epidemiologische Profil eines Infektionsgeschehens zu berücksichtigen ist.

Seit Beginn der COVID-19-Pandemie im Frühjahr 2020 wird die Rolle der Kinder und Kindertageseinrichtungen im Infektionsgeschehen diskutiert. Dem Interesse nach öffentlich unterstützter Bildung, Betreuung und Erziehung von Kindern im Kitaalter steht das berechtigte gesundheitliche Interesse nach einer Eindämmung der weiteren Ausbreitung von SARS-CoV-2-Infektionen gegenüber. Auch Kitabeschäftige sollten vor möglichen Ansteckungen geschützt werden.

### Mögliche Eindämmungsmaßnahmen in Kitas im Verlauf der COVID-19-Pandemie

Die möglichen Eindämmungsmaßnahmen im Feld der frühkindlichen Bildung, Betreuung und Erziehung sind struktureller, organisatorischer oder individueller Art. Eine strukturelle Maßnahme ist die bedeutsame Einschränkung oder das komplette Einstellen des Betreuungsangebots. Weitere Maßnahmen sind in Tab. 1 aufgeführt.

**Table 1:** 

Strukturelle Maßnahmen	(1) Schließungen von Kitas
	(2) Einschränkung des Betreuungsangebots
Organisatorische und individuelle Maßnahmen	(1) *Maßnahmen zur Reduzierung von Kontaktmöglichkeiten in Kitas*
	Bildung fester Kindergruppen und Trennung dieser im Innen- und Außenbereich
	Feste Zuweisung von Personal zu Kindergruppen
	Trennung von Laufwegen in der Einrichtung
	(2) *Maßnahmen zur Reduzierung des Risikos einer Tröpfchen‑/Aerosolübertragung*
	Lüften
	Distanz von Beschäftigten und Kindern innerhalb und zwischen den festen Gruppen
	Tragen einer Mund-Nasen-Bedeckung
	COVID-19-Impfung der Beschäftigten
	(3) *Maßnahmen zur Reduzierung des Risikos einer Kontaktübertragung*
	Händewaschen der Kinder und des Personals
	Desinfektion
	(4) *Maßnahmen zum Umgang mit Symptomen*
	Temperaturmessen bei Kindern und Beschäftigten
	Testen
	Bedingungen festlegen, nach denen der Wiedereintritt in die Kita nach einschlägigen Erkrankungssymptomen zulässig ist

Für die Bereitstellung einer bedarfsgerechten Kinderbetreuung sind nach dem föderalen Prinzip die Bundesländer zuständig ebenso für die Entscheidung einer pandemiebedingten Schließung oder Wiederöffnung. In der COVID-19-Pandemie wurde nach einer ersten allgemeinen Schließung im März 2020 mit Beschluss der Jugendministerkonferenz [12] im April 2020 eine schrittweise Öffnung beschlossen, die von den Ländern in jeweils unterschiedlichem Tempo bis zum Sommer 2020 umgesetzt wurde [13]. Während der sogenannten zweiten und anfänglich auch während der dritten Welle der Pandemie in Deutschland im Herbst/Winter 2020/2021 war die Umsetzung der Lockdownmaßnahmen vergleichbar divers [14]. Auch wenn die Einrichtungen sehr früh neue Hygienekonzepte entwickelten und umsetzten, ist unklar, in welchem Umfang diese Maßnahmen realisiert wurden.

Mit der Verfügbarkeit von wirksamen Impfstoffen wurde den Fachkräften ab Frühjahr 2021 priorisiert ein Impfangebot gemacht. Es gibt bis Juni 2021 keine bundeseinheitlichen Zahlen zur Durchimpfungsrate der Fachkräfte.

### Infektiosität von Kindern

Es gibt nur wenige aussagekräftige Studien, die untersucht haben, wie ansteckend Kinder mit einer SARS-CoV-2-Infektion sind. Eine Veröffentlichung aus Australien fasste die Erfahrungen aus 10 Kitas zusammen, bei denen es zu Expositionen mit SARS-CoV‑2 kam. In nur 3 Kitas war der Primärfall ein Kind, dabei kam es in keiner Kita zu einem Sekundärfall, d. h. zu einer Übertragung des Virus auf eine Kontaktperson [15]. Ob sich die Infektiosität von Kindern verschiedenen Alters unterscheidet, bleibt unklar. Eine dänische und eine südkoreanische Studie, die in dieser Hinsicht fundierte Aussagen zuließen, kamen zu gegenläufigen Ergebnissen [16, 17]. Einer Übersichtsarbeit von Spielberger et al. zufolge steckten Kinder mit SARS-CoV‑2 im Schnitt 13,4 % ihrer Kontaktpersonen an [18], wohingegen jüngere Metaanalysen zu SARS-CoV‑2, bei welchen jeweils Kinder als Indexfall registriert worden sind, Übertragungsraten von 4 % und (ca.) 5 % berichten [19, 20]. Diese Ergebnisse lassen sich nur begrenzt auf Infektionsgeschehen in Kindertageseinrichtungen und die dort betreute Altersgruppe der ca. 0- bis 5‑Jährigen anwenden, da sich die Analysen auf weiter gefasste Altersgruppen beziehen (0–9 Jahre und älter) und die dort verwendeten Daten in erster Linie aus Haushaltsstudien stammen. Die Datengrundlage zur Infektiosität von Kindern in Kitas ist daher immer noch unbefriedigend.

Es gibt Hinweise, dass die Übertragbarkeit durch die von Januar bis etwa Juli 2021 in Deutschland zirkulierende besorgniserregende Variante B.1.1.7 (Alphavariante, Hochplateauphase um etwa 80–90 % im März bis Mai 2021) in allen Altersgruppen höher ist als bei den vorher zirkulierenden Stämmen [21]. Auch eine dänische und eine deutsche Studie kamen auf der Basis von Haushaltsuntersuchungen zu dem Ergebnis, dass die Infektiosität von B.1.1.7-infizierten Kindern im Kitaalter im Vergleich zu den vorher zirkulierenden Varianten angestiegen ist [22, 23].

### Fragestellung

Kitas spielen eine wichtige gesellschaftspolitische Rolle; gleichzeitig begünstigt das durch engen Kontakt geprägte Zusammensein der Kinder untereinander und mit Beschäftigten die Übertragung von Infektionskrankheiten. Aus dieser Konstellation heraus ergeben sich in der COVID-19-Pandemie verschiedene Forschungsfragen. Diese sind vielschichtig und umfassen nicht nur infektionsepidemiologische Aspekte. Im Zuge von längerfristigen Schließungen von Kinderbetreuungen und umfangreichen hygienischen Maßnahmen in den Einrichtungen kommen auch Fragen z. B. zur Belastungssituation von Kitabeschäftigten, Kindern und Eltern hinzu.

Im Juni 2020 wurde eine interdisziplinäre Studie initiiert, in der pädagogische und infektionsepidemiologische Themen zur COVID-19-Pandemie gemeinsam bearbeitet werden. Es wurden unter anderem folgende Fragestellungen adressiert:

1. Wie häufig sind SARS-CoV-2-Infektionen bei Kindern im Kitaalter?
2. Wie häufig waren Kitas in Deutschland im Laufe der Pandemie von SARS-CoV-2-Fällen und infektionsbedingten Schließungen betroffen?
3. Welche Rolle spielten Kinder und Beschäftigte im Infektionsgeschehen in Kitas?
4. Wie wurden COVID-19-bezogene Schutz- und Hygienemaßnahmen in Kitas umgesetzt?

Für die vorliegende Publikation werden Ergebnisse dieser „Corona-KiTa-Studie“ ausgewählt, die sich primär auf die Ebene der Kitaeinrichtung beziehen. Die ebenfalls untersuchte Ebene der betroffenen Familien und Haushalte wurde für die vorliegende Publikation nicht berücksichtigt, um den Umfang des Artikels in einem vertretbaren Rahmen zu halten.

## Methodik

### Corona-KiTa-Studie

**Figure 1:**
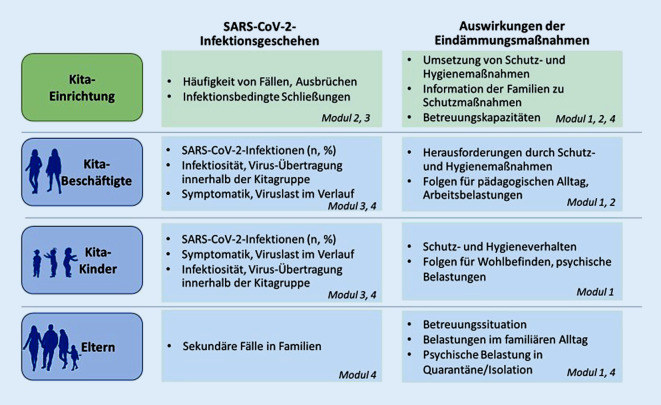

Die Analyse des Infektionsgeschehens in Kitas und die Auswirkungen der Pandemie mit ihren Eindämmungsmaßnahmen erfordern ein vielschichtiges Vorgehen. Zu betrachten sind dabei verschiedene Ebenen: die Ebene der Kitaeinrichtung (z. B. hinsichtlich Hygienemaßnahmen) sowie die Ebene der beteiligten Personengruppen, die neben den Kitakindern auch die Beschäftigten und die Eltern umfasst (Abb. 1).

Die Corona-KiTa-Studie ist ein Kooperationsprojekt zwischen dem Deutschen Jugendinstitut (DJI) und dem Robert Koch-Institut (RKI) und wird vom Bundeministerium für Gesundheit sowie vom Bundesministerium für Familie, Senioren, Frauen und Jugend gefördert. Gemeinsam untersuchen die beiden Institute, unter welchen Bedingungen die Kindertagesbetreuung während der COVID-19-Pandemie angeboten wird, welche Herausforderungen sich aus der Pandemie für die Kitas, deren Personal und die beteiligten Familien ergeben und welche Rolle die Kindertagesbetreuung für die Verbreitung von SARS-CoV‑2 spielt. Diese Fragen werden kooperativ in 4 Modulen bearbeitet:

- Modul 1 (DJI): Befragung von Kitaleitungen, pädagogischem Personal und Eltern zu Herausforderungen im Pandemiealltag,
- Modul 2 (DJI): KiTa-Register mit wöchentlicher Onlinebefragung von Kitas und Kindertagespflegestellen zu Kapazitäten, Schließungen und Hygienemaßnahmen,
- Modul 3 (RKI): kontinuierliche Analyse der amtlichen Meldedaten zur Surveillance der SARS-CoV-2-Fälle bei Kindern (0–5 Jahre) sowie der Ausbruchsgeschehen in Kitas,
- Modul 4 (RKI): anlassbezogene Untersuchungen in Kitas mit akutem SARS-CoV-2-Ausbruchsgeschehen.

Im Folgenden werden Teilergebnisse aus den Modulen 2, 3 und 4 beschrieben.

### KiTa-Register (Modul 2)

Alle Kitas und Kindertagespflegestellen in Deutschland wurden im August 2020 durch zweimaliges postalisches Anschreiben, Presseaufrufe sowie über Trägerverbände und Gewerkschaften aufgerufen, sich für die Teilnahme an einem KiTa-Register anzumelden und an wöchentlichen onlinebasierten Abfragen (Dauer: ca. 5 min) zu beteiligen. Die standardisierten Erhebungen sollen dazu beitragen, die aktuellen Pandemieauswirkungen auf den Alltag der Kindertagesbetreuung zeitnah zu monitoren. Es wird erfasst, ob einzelne Gruppen oder Einrichtungen aufgrund von COVID-19-Erkrankungen oder -Verdachtsfällen geschlossen werden müssen. Seit September 2020 machen die Teilnehmenden wochenaktuelle Angaben zu Personalsituation, Erkrankungsgeschehen und Hygienemaßnahmen [24]. Bis Mai 2021 hatten sich 11.238 Kitas für das Register angemeldet. Dies entspricht etwa 20 % aller in Deutschland gemeldeten Einrichtungen. 8692 Kitas hatten mindestens einmal an einer der wöchentlichen Erhebungen teilgenommen; pro Woche beantworteten ca. 57 % der registrierten Kitas jeweils den Onlinefragebogen (zuletzt ca. 6400). Die teilnehmenden Einrichtungen stimmten hinsichtlich der zentralen Merkmale regionale Verteilung, Träger der Einrichtungen und deren Größe weitgehend mit der Gesamtstichprobe aller Kindertageseinrichtungen überein (gemäß Daten der amtlichen Kinder- und Jugendhilfestatistik).

### Meldedaten (Modul 3)

Die Gesundheitsämter übermitteln gemäß Infektionsschutzgesetz (IfSG) alle labordiagnostisch bestätigten SARS-CoV-2-Infektionen ans RKI. Diese amtlichen Meldedaten werden gezielt für Kinder im Alter von 0–5 Jahren ausgewertet. Ziel dieser Analysen ist die Surveillance der Häufigkeit von SARS-CoV-2-Infektionen bei Kindern im Kitaalter im Vergleich zu den Altersgruppen älterer Kinder und Erwachsener. Dafür wird die Inzidenz pro Meldewoche berechnet und Veränderungen der Werte über die Zeit beobachtet, um ein Ansteigen oder Absinken der Inzidenzen zu erfassen. Die Reihenfolge des Inzidenzanstiegs der Altersgruppen ist ein Hinweis dafür, welche Altersgruppe im Infektionsgeschehen eine führende Rolle einnimmt.

Darüber hinaus werden die ans RKI übermittelten Ausbrüche (Fallhäufungen mit mindestens 2 epidemiologisch verknüpften SARS-CoV-2-Fällen) analysiert, um das Infektionsgeschehen im Kitasetting abzubilden. In der Meldesoftware können Fallhäufungen als Ausbruch angelegt werden und es kann das Infektionsumfeld (z. B. Kindergarten/Hort, privater Haushalt) angegeben werden, in dem sich das Ausbruchsgeschehen ereignete. In der folgenden Auswertung wurden Ausbrüche berücksichtigt, für die als Infektionsumfeld „Kindergarten/Hort“ angegeben wurde. Die Ausbruchshäufigkeit wurde im zeitlichen Verlauf bestimmt. Zudem wird das Alter der involvierten Fälle analysiert, um festzustellen, wie viele Kinder bzw. Erwachsene jeweils pro Ausbruch betroffen sind.

### Anlassbezogene Untersuchungen in Kitas (Modul 4)

Ziel ist es, aufgrund eines konkreten Ausbruchs (Anlass) das SARS-CoV-2-bedingte Infektionsgeschehen bei Kindern und Beschäftigten in Kitas zu untersuchen (fallbezogenes Studiendesign). Ab 10/2020 wurden bundesweit 30 SARS-CoV-2-Kitaindexfälle (d. h. der erste Fall, der dem Gesundheitsamt bekannt geworden ist) und deren enge Kontaktpersonen aus der Kitagruppe in die Studie aufgenommen. Indexfälle konnten dabei sowohl Kitakinder als auch Personal sein. Zum Zeitpunkt der Manuskripterstellung waren die Daten von 28 Kitaausbrüchen aus dem Zeitraum 10/2020–05/2021 verfügbar und analysiert.

Kitakinder und -beschäftigte wurden dabei 4–6 Tage nach Bekanntwerden eines SARS-CoV-2-Falles (Indexfall) in der Einrichtung zu Hause aufgesucht, um weitere Übertragungen von SARS-CoV‑2 durch das infizierte Kitakind (oder den/die Kitabeschäftigte/*n*) festzustellen [25]. Es erfolgten Hausbesuche, weil beim Auftreten eines SARS-CoV-2-Falls in der Regel alle Kinder und Beschäftigten der betroffenen Kitagruppe als enge Kontaktpersonen galten und für sie Isolation bzw. Quarantäne angeordnet wurde. In diesem Rahmen entnahm geschultes Feldpersonal Proben bei den engen Kontaktpersonen des Indexfalls. Die Probengewinnung war bei Kindern und Erwachsenen gleich und bestand aus einem kombinierten Mund-Nase-Abstrich (kein tiefer Rachenabstrich) sowie der Entnahme von Speichel, sodass in der Regel pro Person 2 Proben vorlagen. Die Erwachsenen wurden zudem darin eingewiesen, Proben bei sich selbst bzw. bei ihren Kindern zu entnehmen; sie erhielten hierfür die entsprechenden Materialien. Im Anschluss wurden im Abstand von je 3 Tagen (Tag 3, 6, 9 und 12 nach dem Hausbesuch) 4 Selbstbeprobungen (jeweils Mund-Nase-Abstrich und Speichel) durchgeführt und die gewonnenen Proben an das RKI gesandt. Alle Proben wurden mittels Realtime-Reverse-Transkriptase (rRT-PCR) auf SARS-CoV-2-RNA untersucht. So konnten neu auftretende SARS-CoV-2-Infektionen bei engen Kontaktpersonen der infizierten Kitakinder bzw. -beschäftigen schnell identifiziert werden. Der Anteil an Folge- oder Zweitinfektionen (sekundäre Fälle) in der Kita wird als prozentualer Wert bezogen auf die Kontaktpersonen berechnet und beschreibt die Übertragbarkeit des Virus ausgehend von einem Indexfall (sogenannte sekundäre Attack-Rate oder sekundäre Übertragungsrate).

### Orientierung am Pandemiegeschehen

Das Infektionsgeschehen und die Eindämmungsmaßnahmen in Kitas werden auch im zeitlichen Verlauf beobachtet. Dabei sind für die Kitas relevante Ereignisse in Tab. 2 aufgeführt; auf sie wird bei der Präsentation der Daten Bezug genommen. Die Auswertungen der vorliegenden Publikation beziehen sich auf den Zeitraum bis Ende Mai 2021, jeweils beginnend von März 2020 (Meldedaten, Modul 3) bzw. August 2020 (KiTa-Register, Modul 2) bzw. Oktober 2020 (Modul 4, anlassbezogene Untersuchungen).

**Table 2:** 

Zeitpunkt/Zeitraum	Ereignis	Erläuterung
11.03.2020	Beginn der COVID-19-Pandemie	Die WHO erklärt das weltweite SARS-CoV-2-Infektionsgeschehen zu einer Pandemie
März–Mai 2020	Erste COVID-19-Welle	Höchste Inzidenz am 02.04.2020 mit 6550 Neuerkrankungen/Tag
15.03.2020	Kitaschließungen	In Deutschland werden Kitas und Schulen erstmals geschlossen
23.03.2020	Erster Lockdown	Bundesweiter Lockdown mit umfassenden Kontaktbeschränkungen und weitreichenden Schließungen, u. a. der Gastronomie
20.04.2020	Erste Lockerungen	Einkaufen in größeren Geschäften wieder erlaubt, teilweise Wiederaufnahme des Schulbetriebs etc.
Mitte Oktober–Ende Februar 2021	Zweite COVID-19-Welle	Höchste Inzidenz am 23.12.2020 mit 33.991 Neuerkrankungen/Tag
02.11.2020	Beginn des „Teillockdowns“/Lockdowns light	Verschärfte Kontaktbeschränkungen, Schließung Gastronomie- und Tourismusbranche
16.12.2020	Beginn des zweiten „harten“ Lockdowns	Kita- und Schulschließungen
27.12.2020	Impfbeginn in Deutschland	–
Januar 2021	Beginn der Ausbreitung der B.1.1.7-Mutation (Alphavariante) in Deutschland	Anteil der Alphavariante Ende Januar: 6 % aller nachgewiesenen Infektionen
März 2021	Beginn der Impfung von Erzieher/innen	Änderung der Impfpriorisierung im Februar 2021
08.03.2021	Geänderte Teststrategie	Kostenlose „Bürgertests“ erhältlich
März 2021	Beginn der dritten COVID-19-Welle	Höchste Inzidenz am 21.04.2021 mit 29.472 Neuerkrankungen/Tag
März 2021	Alphavariante ist vorherrschender Virusstamm in Deutschland	März–Mai 2021: Hochplateauphase der B.1.1.7-Mutation (Alphavariante) von 80–90 %

## Ergebnisse

### Wie häufig traten SARS-CoV-2-Infektionen bei Kindern im Kitaalter auf?

#### Ergebnisse aus den COVID-19-Meldedaten

**Figure 2:**
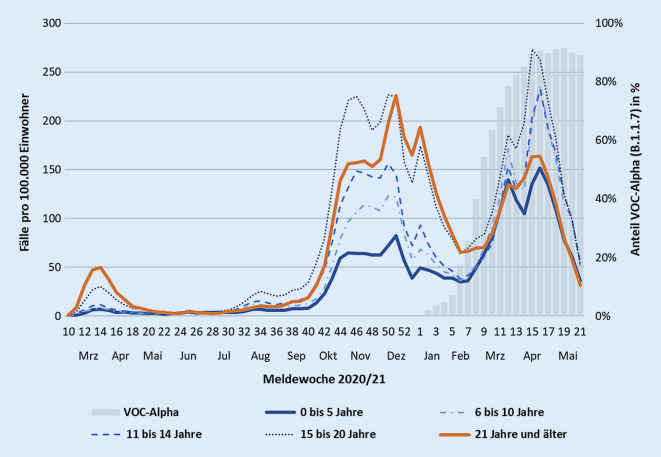

In der ersten Welle im Frühjahr 2020 sowie in der zweiten Welle im Herbst 2020 stieg die Inzidenz bei den 0‑ bis 5‑jährigen Kindern im Vergleich zur Inzidenz der Erwachsenen (> 20 Jahre) zeitversetzt jeweils erst 1–2 Wochen später an. Damit folgten die Infektionszahlen bei Kindern im Kitaalter also zeitlich der Situation in der Gesamtbevölkerung und gingen ihr nicht voraus (Abb. 2). Im Gegensatz dazu stiegen die Inzidenzen bei beiden Altersgruppen während der dritten Welle im Frühjahr 2021 synchron an. Dieses Geschehen fand gleichzeitig mit dem Anstieg des Anteils der Alphavariante statt.

In der ersten Welle war die Inzidenz der Erwachsenen teilweise 7‑ bis 9‑mal höher als die der Kinder im Kitaalter. Während des Peaks in der zweiten Welle verringerte sich dieses Verhältnis auf den Faktor 3 bis 4. In der dritten Welle blieb die Inzidenz der Erwachsenen deutlich unter dem Niveau der zweiten Welle, während die Inzidenz der Kinder im Kitaalter das Niveau vom Herbst 2020 deutlich überstieg.

**Figure 3:**
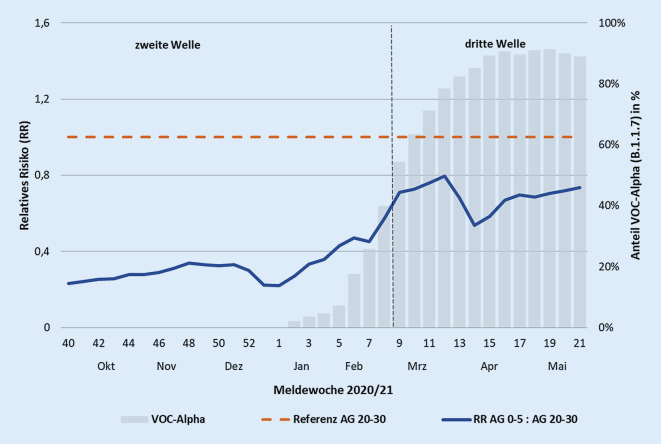

Das veränderte Verhältnis der Infektionszahlen von Kindern im Alter von 0 bis 5 Jahren zu Erwachsenen wird auch bei der Betrachtung des relativen Risikos (RR) ersichtlich (Abb. 3); dieses bewertet die Inzidenz der 0–5 Jahre alten Kinder relativ zur Inzidenz der 20–30 Jahre alten Erwachsenen. Als Referenz wurde die Altersgruppe 20–30 Jahre gewählt, da in dieser Gruppe bis Ende Mai 2021 von einer niedrigen Impfquote ausgegangen werden konnte. Das relative Risiko, dass ein Kind als SARS-CoV-2-Fall gemeldet wird, war im Verlauf der zweiten Welle relativ stabil und nahm ab Beginn der dritten Welle kontinuierlich zu (RR-Anstieg von etwa 0,3 auf 0,8).

### Wie häufig waren Kitas von SARS-CoV-2-Fällen und infektionsbedingten Schließungen betroffen?

#### Ergebnisse aus dem KiTa-Register

Im KiTa-Register wurden zwischen 08/2020 und 05/2021 insgesamt 2094 Schließungen ganzer Einrichtungen und zusätzlich 6334 Gruppenschließungen für diesen Zeitraum gemeldet, 570 Kitas meldeten mehrere Einrichtungs- und 1695 Kitas mehrere Gruppenschließungen. Kindertageseinrichtungen waren davon deutlich häufiger betroffen als Kindertagespflegestellen.

**Figure 4:**
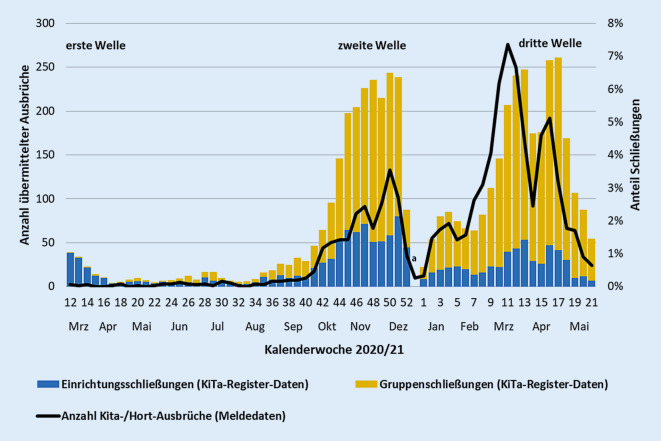

Abb. 4 zeigt die dokumentierten Schließungen im Zeitverlauf. Anhand des Anstiegs der Schließungen lassen sich unschwer die Konsequenzen der zweiten und dritten Welle für die Kitas erkennen. Etwa mit Beginn des Kitajahres 2020/2021 (September 2020) zeichnete sich ab, dass im Fall von notwendigen Schließungen zunehmend nur eine der Gruppen und nicht die gesamte Einrichtung geschlossen wurde.

#### Ergebnisse aus den COVID-19-Meldedaten

Seit Beginn der Pandemie wurden bis Ende Mai 2021 insgesamt 3286 Ausbrüche mit Infektionsumfeld „Kindergarten, Hort“ übermittelt, denen 20.946 Fälle zugeordnet wurden. Während in der ersten Welle nur sporadisch Ausbrüche in Kitas bzw. Horteinrichtungen übermittelt wurden (sie waren auch größtenteils geschlossen), zeichneten sich die zweite und besonders die dritte Welle deutlich im Ausbruchsgeschehen ab (Abb. 4). Der Anstieg der Ausbruchshäufigkeit und der damit verbundenen Fälle ist dabei recht ähnlich mit dem Inzidenzverlauf, vor allem der erwachsenen Bevölkerung. Insbesondere ab 02–03/2021 gab es einen steilen Anstieg, der das Niveau vom Herbst 2020 deutlich überstieg. Dieser Effekt fällt zeitlich mit der Ausbreitung der Alphavariante zusammen. Auffällig ist, dass die Zahl der Kitaausbrüche in der dritten Welle diejenige in der zweiten Welle um etwa das Doppelte überstieg. Rückgänge im Ausbruchsgeschehen lassen sich in den Zeiträumen der Ferien erkennen, z. B. in den Weihnachtsferien (12/2020–01/2021) und den Osterferien (03/2021). Im Mai 2021 befand sich die Zahl der Ausbrüche wieder auf einem niedrigen Niveau.

### Welche Rolle spielten Kinder und Beschäftigte im Infektionsgeschehen in Kitas?

#### Ergebnisse aus den COVID-19-Meldedaten

Größere Ausbrüche im Kita‑/Hortsetting waren die Ausnahme. Im Median kam es zu 4 COVID-19-Fällen pro Ausbruch, wobei sich die mediane Ausbruchsgröße 02–03/2021 vorübergehend auf 5–6 Fälle erhöhte. In 85 % aller gemeldeten Kita‑/Hortausbrüche waren auch Kinder Teil des Ausbruchs. Im Verlauf der dritten Welle nahm der Anteil der 0‑ bis 5‑Jährigen in Kita‑/Hortausbrüchen (im Vergleich zu Erwachsenen bzw. älteren Kindern) stetig zu: von 35 % in der zweiten Welle im Dezember 2021 auf etwa 46 % Ende Mai 2021. Zeitlich parallel begannen die Ausbreitung der Alphavariante sowie das Impfangebot für das Kitapersonal.

#### Zwischenergebnisse aus den anlassbezogenen Untersuchungen

Eine vorläufige Auswertung der ersten 28 Kitas aus dem Zeitraum 10/2020 bis 05/2021 zeigt, dass die Infektionsgeschehen in den untersuchten Kitas sehr unterschiedlich sind. Es wurden, ausgehend vom jeweiligen Indexfalll, oftmals keine oder nur vereinzelte Übertragungen (sekundäre Fälle) bei den Teilnehmenden aus der Kitagruppe gefunden (Tab. 3). In einer einzelnen Kita konnten jedoch bei 13 Personen der Kitagruppe weitere Ansteckungen festgestellt werden.

**Table 3:** 

Indexfall	Nummer der Kitagruppe chronologisch	Monat der Rekrutierung	Untersuchte Kontaktpersonen in Kitas (*n*)	Sekundäre Fälle (*n*)	Sekundäre Attack-Rate (%)	Nachweis Virusvariante
Kind	1	10/20	13	0	0	–
	4	11/20	15	1	6,7	–
	5	12/20	12	0	0	–
	7	01/21	14	1	7,1	–
	8	01/21	7	0	0	–
	12	02/21	9	0	0	Alpha
	14	02/21	5	0	0	–
	15	02/21	16	1	6,3	Alpha
	16	02/21	18	0	0	Alpha
	17	03/21	7	0	0	Alpha
	19	03/21	17	1	5,9	Alpha
	21	03/21	9	0	0	–
	22	03/21	8	0	0	Alpha
	25	04/21	7	0	0	Alpha
	26	04/21	19	8	42,1	Alpha
	*Gesamt*	*–*	*176*	*12*	*6,8*	*–*
Personal	2	10/20	9	1	11,1	–
	3	11/20	18	0	0	–
	6	01/21	23	0	0	–
	9	01/21	8	0	0	–
	10	02/21	12	2	16,7	–
	11	02/21	12	5	41,7	Alpha
	13	02/21	10	0	0	–
	18	03/21	7	0	0	–
	20	03/21	13	1	7,7	–
	23	03/21	14	4	28,6	Alpha
	24	03/21	16	13	81,3	Alpha
	27	05/21	2	0	0	Alpha
	28	05/21	7	0	0	Alpha
	*Gesamt*	*–*	*151*	*26*	*17,2*	*–*

In den ersten 28 Kitas wurden insgesamt 38 Kontaktpersonen positiv auf SARS-CoV‑2 getestet (12 Kinder und 26 Erwachsene; Tab. 3). In den Kitas, in denen der Indexfall ein Kind war, konnte eine Übertragung von SARS-CoV‑2 auf 0–42 % der Kontaktpersonen in der Kitagruppe beobachtet werden (durchschnittlich 6,8 %, insgesamt 12/176 Kontaktpersonen infiziert). In den Kitas, in denen der Indexfall ein Erwachsener war, wurde eine Übertragung auf 0–81 % der Kontaktpersonen beobachtet (durchschnittlich 17,2 %, insgesamt 26/151 Kontaktpersonen infiziert, Unterschied nicht signifikant). Der hohe Durchschnittswert ist hauptsächlich auf einen Kitaausbruch mit einem erwachsenen Indexfall zurückzuführen, bei dem 13 Übertragungen festgestellt wurden; bei Eliminierung dieses Ausreißers liegt die durchschnittliche sekundäre Attack-Rate durch Erwachsene niedriger (9,6 %).

Kinder in der Kitagruppe haben sich tendenziell seltener mit SARS-CoV‑2 angesteckt als Beschäftigte: 10,3 % aller kindlichen Kontaktpersonen infizierten sich (Indexfall Kind oder Erwachsener) und 16,0 % aller erwachsenen Kontaktpersonen (der Unterschied ist allerdings nicht statistisch signifikant). Von den sekundären Übertragungen wurden 89 % beim ersten Hausbesuch entdeckt, 11 % in den darauffolgenden Selbstbeprobungen.

Die genannten Zwischenergebnisse sind vorläufig; die derzeitige Stichprobe beruht auf einem Teil der untersuchten Ausbrüche und aktuell wird bei komplexeren Ausbrüchen versucht, das Infektionsgeschehen zu rekonstruieren, sodass sich noch Änderungen ergeben können.

### Wie wurden COVID-19-bezogene Schutz- und Hygienemaßnahmen in Kitas umgesetzt?

#### Ergebnisse aus dem KiTa-Register

Von Herbst 2020 bis Winter 2020/2021 zeigten sich die Einrichtungen sehr engagiert, die Gruppenräume regelmäßig zu lüften und die Oberflächen zu desinfizieren. Entsprechende Zustimmungswerte für „Lüften“ fielen zu keinem Zeitpunkt unter 95 % aller in der jeweiligen Woche antwortenden Einrichtungen. Die Angabe zu den „Oberflächendesinfektionen“ pendelte jeweils um 90 % der Einrichtungen. Eine eher unübliche Maßnahme war ab Beginn der Erhebung und über den gesamten Verlauf des Winters das Temperaturmessen bei Beschäftigten und Kindern (Angaben sehr stabil bei etwa 7 % und 4 % aller jeweils antwortenden Einrichtungen). Etwas mehr Variation zeigte sich im Anteil der Einrichtungen, die das Gruppenkonzept anpassten und feste Kindergruppen bildeten oder wieder auflösten. Nach anfänglicher fester Gruppenbildung im Frühjahr 2020 wechselten im Verlauf des Herbsts und Winters 2020/2021 immer wieder Einrichtungen zu offeneren Gruppenkonzepten und ggf. bei ansteigender zweiter und dritter Welle auch wieder in stärker geschlossene Kindergruppen.

## Diskussion

### Zusammenfassung und Einordnung der wichtigsten Ergebnisse

Das SARS-CoV-2-Infektionsgeschehen in Kitas bzw. bei Kindern im Kitaalter war während des Pandemieverlaufs von Frühjahr 2020 bis Mai 2021 so dynamisch wie die epidemiologische Gesamtsituation. Insbesondere in der zweiten und vor allem in der dritten Pandemiewelle (Herbst 2020 und Frühjahr 2021) stieg die Häufigkeit der SARS-CoV-2-Ausbrüche in Kitas deutlich an, begleitet von einer erheblichen Zunahme an Kita- und Gruppenschließungen. Bedeutsam sind die Veränderungen im zeitlichen Verlauf: So scheint sich die Rolle der Kitakinder in der dritten Welle geändert zu haben. Das Infektionsgeschehen bei Kindern im Kitaalter war in den ersten 2 Wellen weniger stark ausgeprägt als bei Älteren und Inzidenzen stiegen zeitversetzt nach denjenigen von älteren Kindern und Erwachsenen an. Bei SARS-CoV-2-Ausbrüchen in Kitas erhöhte sich hingegen in der dritten Welle der Anteil betroffener Kinder im Alter von 0 bis 5 Jahren an allen Infektionsfällen kontinuierlich (von 35 % auf 46 %), während der Anteil an betroffenen Erwachsenen abnahm. Die Inzidenz bei Kindern in der dritten Welle überstieg diejenige der zweiten Welle deutlich, während die Inzidenz der Erwachsenen sich in beiden Wellen in etwa die Waage hielt und der bisher beobachtete zeitliche Verzug des Inzidenzanstiegs zwischen Kitakindern und Erwachsenen ausblieb. Das lässt sich vermutlich nicht alleine mit einer zunehmenden Impfquote bei Älteren erklären, da auch im Vergleich zu den 20- bis 30-jährigen Erwachsenen (die aufgrund der Priorisierung in der Coronavirusimpfverordnung vermutlich relativ wenig geimpft waren) das relative Risiko der 0‑ bis 5‑jährigen Kinder für eine SARS-CoV-2-Infektion in der dritten Welle deutlich anstieg. Diese Beobachtungen sind ein Hinweis darauf, dass die Alphavariante die Bedeutung der Kinder im Infektionsgeschehen im Vergleich zur zweiten Welle vergrößert hat. Andere Einflussfaktoren sind ebenfalls denkbar. So kann auch die im Laufe der Zeit geänderte Teststrategie eine Rolle spielen, bei der u. a. Reihentestungen von asymptomatischen Kontaktpersonen oder von Einreisenden angeordnet sowie sukzessive auch im Kitaumfeld Testangebote geschaffen wurden. Daten aus der laborbasierten SARS-CoV-2-Surveillance zeigen, dass ab Herbst 2020 deutlich mehr Kinder mittels eines PCR-Tests getestet wurden, und auch der Anteil positiv auf SARS-CoV‑2 getesteter Kinder bewegte sich auf einem höheren Niveau.

Die anlassbezogenen Untersuchungen in 28 Kitas mit akutem SARS-CoV-2-Fall zeigten in ersten Auswertungen, dass bei kindlichen Indexfällen im Durchschnitt nur ein Bruchteil (ca. 7 %) der untersuchten Kontaktpersonen aus der betroffenen Kitagruppe angesteckt wurde. Allerdings unterschieden sich die Übertragungshäufigkeiten zwischen einzelnen Kitas zum Teil deutlich.

Die Kitas bzw. die verantwortlichen Gesundheitsämter reagierten etwa ab Herbst 2020 häufiger mit Gruppenschließungen statt mit Schließungen ganzer Einrichtungen, was mit einer besseren Umsetzung getrennter Gruppenkonzepte zusammenhängen kann. Insbesondere scheinen die Kitas diese Maßnahmen stärker an das Infektionsgeschehen angepasst zu haben, während die grundlegenden Eindämmungsmaßnahmen wie Lüften und Desinfizieren über die gesamte Pandemie hinweg kontinuierlich von fast allen Kitas dokumentiert wurden.

### Stärken und Limitationen der Studie

Das Zusammenführen verschiedener Datenquellen und Disziplinen ist eine Stärke der Studie und trägt dazu bei, die komplexen und vielfältigen Auswirkungen der COVID-19-Pandemie auf Kitas entsprechend vielschichtig abzubilden. So gibt die Auswertung der Meldedaten einen guten Hinweis auf den Verlauf der Häufigkeit von SARS-CoV-2-Ausbrüchen im Kita‑/Hortsetting. Das korrespondiert aber nicht immer mit den gesellschaftspolitischen Auswirkungen wie Schließungen einer Kita(-gruppe), da diese Schließungen auch bei einzelnen SARS-CoV-2-Fällen, bei Verdachtsfällen oder auch bei COVID-19-bedingtem Personalmangel passieren können. Ebenso können die Meldedaten über Veränderungen der Inzidenzwerte bei Kindern im Kitaalter Hinweise auf deren Rolle im Infektionsgeschehen geben; die Untersuchung der Übertragungen durch SARS-CoV-2-infizierte Kitakinder im Ausbruchsgeschehen ergänzt diese Beobachtungen durch konkrete Angaben zur Infektiosität, die allerdings nur in einer – im Vergleich mit den Meldedaten – kleinen Stichprobe erhoben wurden.

Eine weitere Stärke der Corona-KiTa-Studie ist der Stichprobenumfang des KiTa-Registers, für das sich etwa 20 % der Kitas in Deutschland angemeldet haben, von denen im Schnitt mehr als die Hälfte an den wöchentlichen Befragungen teilnimmt. Nicht ausgeschlossen werden kann hingegen ein Selektionsbias, z. B. hinsichtlich besonders engagierter und personell gut besetzter Kitas, oder eine soziale Erwünschtheit beim Antwortverhalten zu den Fragen, bei denen es um das Einhalten der Hygiene- und Abstandsregeln geht. Die amtlichen Meldedaten bieten eine Vollerfassung aller laborbestätigten SARS-CoV-2-Fälle; allerdings sind die Daten der 0‑ bis 5‑Jährigen nicht deckungsgleich mit Kindern, die tatsächlich eine Kita besuchen. Für die anlassbezogenen Untersuchungen in Kitas mit einem SARS-CoV-2-Fall kann als limitierender Faktor gelten, dass die Teilnahme an der Studie freiwillig war und nicht immer alle Personen einer Kitagruppe an der Studie teilnahmen. Die Testungen begannen aus logistischen Gründen erst 4–6 Tage nach Diagnose des Indexfalls. Einige sekundäre Fälle sind dadurch möglicherweise nicht erkannt oder erfasst worden. Dem wurde dadurch entgegengewirkt, dass die Attack-Rate sich nur auf die teilnehmenden Kontaktpersonen bezieht und dass eng mit den jeweiligen Gesundheitsämtern zusammengearbeitet wurde, um fehlende Informationen zu erhalten. Eine Stärke ist die kontinuierliche Selbstbeprobung im Verlauf nach Quarantänisierung, sodass zusätzliche Übertragungen identifiziert werden konnten.

## Fazit

Die beschriebenen Ergebnisse der Corona-KiTa-Studie zeigen auf, dass das Infektionsgeschehen und auch die Betreuungssituation in Kitas im Laufe einer Pandemie oder Epidemie kontinuierlich und systematisch beobachtet werden sollten (Monitoring), um eine aktuelle Datenbasis für gesundheits- und bildungspolitische Entscheidungen für etwaige Eindämmungsmaßnahmen bereitzustellen. Dafür führte die Studie unterschiedliche Public-Health-Perspektiven zusammen und berichtete monatlich die aktuellen Daten und Analysen. So können verschiedene Belastungen und Bedarfe von Kitas in der COVID-19-Pandemie erfasst werden und als Grundlage für politische Entscheidungen dienen. Die Verknüpfung von Meldedaten aus dem Gesundheits- und Bildungsbereich, Umfragen und individuellen Ausbruchsdaten in der Corona-KiTa-Studie liefert eine solide Datenbasis als Entscheidungsgrundlage. Die Studie kombiniert dabei Bildungs- und Sozialwissenschaften, epidemiologische Biostatistik und Laboruntersuchungen. Durch diese Zusammenarbeit kann ein Mehrwert geschaffen werden, der auch für die Zukunft als ein Beispiel zum Monitoring des Geschehens im Setting Kita während einer Pandemie oder Epidemie dienen könnte. Um auf die nächste Pandemie vorbereitet zu sein, sollte erwogen werden, öffentliche Monitoringsysteme einzurichten, die Gesundheitsmeldedaten mit Daten aus Bereichen der frühkindlichen Bildung, Betreuung und Erziehung kombinieren.
